# Turnover of multiple sex chromosomes in *Harttia* catfish (Siluriformes, Loricariidae): a glimpse from whole chromosome painting

**DOI:** 10.3389/fgene.2023.1226222

**Published:** 2023-07-28

**Authors:** Francisco de Menezes Cavalcante Sassi, Geize Aparecida Deon, Alexandr Sember, Thomas Liehr, Osvaldo Takeshi Oyakawa, Orlando Moreira Filho, Luiz Antonio Carlos Bertollo, Marcelo Ricardo Vicari, Marcelo de Bello Cioffi

**Affiliations:** ^1^ Laboratório de Citogenética de Peixes, Departamento de Genética e Evolução, Universidade Federal de São Carlos, São Carlos, Brazil; ^2^ Laboratory of Fish Genetics, Institute of Animal Physiology and Genetics, Czech Academy of Sciences, Liběchov, Czechia; ^3^ Institut für Humangenetik, Universitätsklinikum Jena, Jena, Germany; ^4^ Museu de Zoologia, Universidade de São Paulo, São Paulo, Brazil; ^5^ Departamento de Biologia Estrutural, Molecular e Genética, Universidade Estadual de Ponta Grossa, Ponta Grossa, Brazil

**Keywords:** microdissection, WCP, chromosomal rearrangements, karyotype, evolution

## Abstract

The remarkable fish biodiversity encompasses also great sex chromosome variability. *Harttia* catfish belong to Neotropical models for karyotype and sex chromosome research. Some species possess one of the three male-heterogametic sex chromosome systems, XY, X_1_X_2_Y or XY_1_Y_2_, while other members of the genus have yet uncharacterized modes of sex determination. Particularly the XY_1_Y_2_ multiple sex chromosome system shows a relatively low incidence among vertebrates, and it has not been yet thoroughly investigated. Previous research suggested two independent X-autosome fusions in *Harttia* which led to the emergence of XY_1_Y_2_ sex chromosome system in three of its species. In this study, we investigated evolutionary trajectories of synteny blocks involved in this XY_1_Y_2_ system by probing six *Harttia* species with whole chromosome painting (WCP) probes derived from the X (HCA-X) and the chromosome 9 (HCA-9) of *H. carvalhoi*. We found that both painting probes hybridize to two distinct chromosome pairs in Amazonian species, whereas the HCA-9 probe paints three chromosome pairs in *H. guianensis,* endemic to Guyanese drainages. These findings demonstrate distinct evolutionary fates of mapped synteny blocks and thereby elevated karyotype dynamics in *Harttia* among the three evolutionary clades.

## 1 Introduction

The Loricariidae family, which includes the Neotropical armored catfishes, is a promising group for evolutionary studies. Slightly above 1,000 species are distributed among 115 genera and most of this diversity occurs in the subfamilies Hypostominae, Hypoptopomatinae, and Loricariinae (Fricke et al., 2023). Loricariids have spread to almost all freshwater environments during their evolutionary history, being thus especially valuable in the artisanal fishery and the ornamental fish trade (Novák et al., 2022). Despite their great diversity in morphology, ecology, and habitats, Loricariidae is recovered as a monophyletic group in both morphological and molecular phylogenetic reconstructions, though the relationships between the tribes and genera have not been fully resolved so far (Covain et al., 2016; Londoño-Burbano and Reis, 2021). In this context, cytogenetic research provides important landmarks for solving issues related to taxonomy, phylogeny, and biodiversity in loricariids (e.g., Rocha-Reis et al., 2018; Glugoski et al., 2020; Takagui et al., 2020; Takagui et al., 2023; de Paula et al., 2022) as well as in other teleosts (Bellafronte et al., 2005; Cioffi et al., 2018; Gavazzoni et al., 2023). Moreover, particularly in the Neotropical region, with the richest freshwater biodiversity on the planet (Albert et al., 2020), cytogenetic investigation has abundant opportunities to deeply scrutinize the relationship between karyotype and sex chromosome evolution and reproductive isolation/diversification (Cioffi et al., 2017; Cioffi et al., 2018; de Souza et al., 2022).

Armored catfishes exhibit a wide variation in diploid chromosome numbers, ranging from 2n = 34 in *Rineloricaria latirostris* (Giuliano-Caetano, 1998) to 2n = 96 in *Hemipsilichthys gobio* (published as *Upsilodus* sp. in Kavalco et al., 2005). Some reports also documented population polymorphisms in the karyotype organization and repetitive DNA distribution, and the presence of B and sex chromosomes, the latter in different stages of differentiation (Scavone and Julio, 1994; Centofante et al., 2006; Rosa et al., 2012; Porto et al., 2014; Glugoski et al., 2018; Marajó et al., 2023). While the plesiomorphic 2n for loricariids has been supposed to be 2n = 54 (Artoni and Bertollo, 2001), this issue is still under intense debate, particularly considering the karyotypic features of species from the early-diverging clades (Takagui et al., 2020; Sassi et al., 2021).

Teleost fishes are well known for remarkable diversity of sex determination mechanisms (Devlin and Nagahama, 2002; Godwin and Roberts, 2018; Shen and Wang, 2018; Guiguen et al., 2019; Schartl et al., 2023). To date, more than 500 species have been found to carry one of nine so far known sex chromosome systems (El Taher et al., 2021; Sember et al., 2021), five of which are so-called multiple sex chromosome systems as they involve more than two chromosomes. Among 81 currently known cases of teleost multiple sex chromosomes (Deon et al., 2020; Sember et al., 2021; Ferchaud et al., 2022; de Araújo et al., 2023; Marajó et al., 2023; Nirchio et al., 2023), only nine are of the XY_1_Y_2_ type and evolved either via Y-fission or X-autosome fusion (Deon et al., 2020; Sember et al., 2021), Intriguingly, three of these systems have been found in *Harttia* spp., all from the Southeastern Brazilian drainages (Centofante et al., 2006; Blanco et al., 2013; Deon et al., 2020).

The genus *Harttia* (Loricariidae, Loricariinae) currently harbors 28 valid species (Oyakawa et al., 2018; Caldas et al., 2022; Fricke et al., 2023) as well as three other predicted species based on the cytogenetic data (Sassi et al., 2021). *Harttia* catfish are organized into three phylogenetic clades grouping representatives from Guyanese, Amazonian, and Southeastern Brazilian drainages (Covain et al., 2016). *Harttia* spp. are excellent models for studying interplay between chromosomal and species diversity in Neotropical fishes as they underwent extensive karyotype repatterning. Their 2n varies from 2n = 52/53 in *H. carvalhoi* (Centofante et al., 2006) to 2n = 62 in *H. absaberi* and *Harttia* sp. 2 (Rodrigues, 2010; Deon et al., 2020) and at least three distinct sex chromosome systems have been described in some species: an XX/XY sex chromosome system in *H. rondoni* (Sassi et al., 2020), and the two following multiple sex chromosome systems: X_1_X_1_X_2_X_2_/X_1_X_2_Y in *H. duriventris*, *H. punctata*, and *H. villasboas* (Blanco et al., 2013; Sassi et al., 2020), and XX/XY_1_Y_2_ in *H. carvalhoi*, *H. intermontana* and *Harttia* sp. 1 (Centofante et al., 2006; Deon et al., 2020). Studies using whole chromosome painting (WCP) probes have recently demonstrated that both the XY and X_1_X_2_Y sex chromosome systems are homologous, while the XY_1_Y_2_ system is formed by different linkage groups and thus has evolved independently (Deon et al., 2022a; b). Intriguingly, the three XY_1_Y_2_-bearing species share the ancestral sex chromosomes but differ by autosomal additions. While *H. carvalhoi* and *Harttia* sp. 1 entirely share the identity of XY_1_Y_2_ chromosomes, the same system in *H. intermontana* involves linkage group corresponding to chromosome 9 in *H. carvalhoi*, which implies two independent X-autosome fusions behind the origin of known *Harttia* XY_1_Y_2_ sex chromosomes (Deon et al., 2022a).

Here, we investigate six *Harttia* species from the Guyanese (*H. guianensis*) and Amazonian (*H. villasboas, H. duriventris, H. rondoni, Harttia* sp., and *H. dissidens*) clades using cross-species (i.e., Zoo-FISH) WCP experiments to examine the trajectory of karyotype and sex chromosome evolution. For this, we used the probes derived from the largest chromosomes (pairs X and 9) of *H. carvalhoi* (2n = 52/53 XY_1_Y_2_), bearing in mind that chromosome 9 is involved in the XY_1_Y_2_ sex chromosome system of *H. intermontana*. The results allowed us to demonstrate distinct evolutionary fates of mapped synteny blocks and thereby elevated karyotype dynamics in *Harttia*.

## 2 Materials and methods

### 2.1 Sampling and chromosome preparation

**FIGURE 1:**
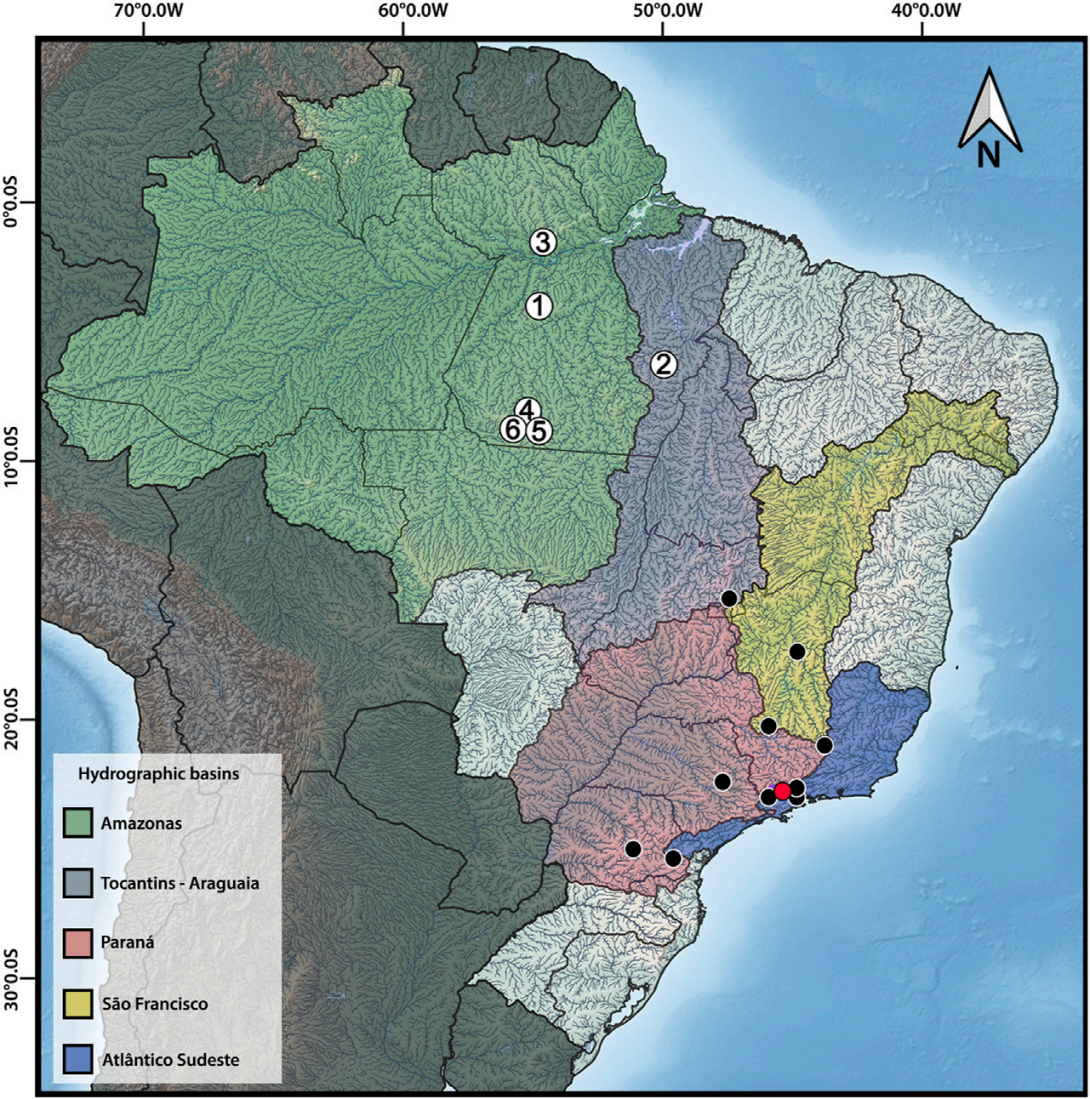
Distribution map of *Harttia* species with available cytogenetic data, highlighting the six species studied herein: 1 – *H. dissidens*, 2 – *H. duriventris*, 3 – *H. guianensis*, 4 – *H. rondoni*, 5 – *H. villasboas*, 6 – *Harttia* sp. 3. The red circle indicates the collection site of *H. carvalhoi*, a species used to construct WCP probes, while the black circles correspond to the distribution of other *Harttia* species which were not included in the present investigation. Major Brazilian hydrographic basins are highlighted: Amazonas in green, Tocantins-Araguaia in purple, Paraná in red, São Francisco in yellow, and Atlântico-Sudeste in blue. The map was created in QGIS 3.20 using Natural Earth package.

Individuals belonging to seven species (including *H. carvalhoi* used solely for the WCP probes preparation) were collected in distinct locations in Amazonas and Tocantins-Araguaia River basins (Figure 1; Table 1), with the approval of the Brazilian environmental agencies ICMBIO/SISBIO (License 48628-14) and SISGEN (A96FF09). We used cells from the posterior kidney and spleen tissue to obtain metaphase chromosomes by the classical air-drying technique (Bertollo et al., 2015). The specimens were deposited in the fish collection of the Instituto Nacional de Pesquisa da Amazônia (INPA) and the Museu de Zoologia da Universidade de São Paulo (MZUSP) as indicated in Table 1. The anesthesia and ethical practices used during the procedures were previously approved by the Ethics Committee on Animal Experimentation of the Universidade Federal de São Carlos (Process number CEUA 1853260315).

**TABLE 1:** *Harttia* species, collection sites, diploid chromosome numbers and sex chromosomes, sampling, and voucher numbers.

Species	Locality	2n	N	Voucher
1 - *Harttia dissidens*	Rurópolis – PA 4°5’37.8’’S 55°0’30.2’’W	54♀♂	07♀, 25♂	INPA-ICT 059577
2 *- H. duriventris*	Canaã dos Carajás – PA 6°30’06.5’’S 50°02’35.5’’W	56♀, 55♂ (X_1_X_2_Y)	08♀, 07♂	MZUSP 126598
3 - *H. guianensis*	Alenquer – PA 1°29’02.2’’S 54°50’31.2’’W	58♀♂	06♀, 10♂	INPA-ICT 059584
4 - *H. rondoni*	Altamira – PA 8°38’53’’S 55°01’41’’W	54♀♂ (XY)	15♀, 14♂	MZUSP 127606
5 - *H. villasboas*	Altamira – PA 8°44’09’’S 54°57’46’’W	56♀, 55♂ (X_1_X_2_Y)	34♀, 38♂	MZUSP 126599
6 - *Harttia* sp. 3	Altamira – PA 08°39’20.7’’S 55°09’24.1’’W	54♀♂	11♀, 15♂	MZUSP 127605

### 2.2 Microdissection and preparation of hybridization probes

We selected the largest metacentric (X) and the largest submetacentric (No. 9) chromosomes of *H. carvalhoi* since both chromosomes are involved in the two variant forms of the XY_1_Y_2_ system in *Harttia* species (Deon et al., 2022b). Glass-needle-based microdissection was used to isolate 15 copies of each target chromosome under an inverted microscope (Zeiss Axiovert 135). The obtained DNA was amplified using a degenerate oligonucleotide-primed polymerase chain reaction (DOP-PCR) following Yang et al. (2009). The probes were labeled with Spectrum Green-dUTP and Spectrum Orange-dUTP (Vysis, Downers Grove, United States), in a secondary DOP-PCR reaction using 1 µL of the first amplification as template (Yang and Graphodatsky, 2009).

### 2.3 Whole chromosome painting (WCP)

Chromosome preparations were aged at 60 °C for 1h, then treated with RNAse solution [1.5 µL RNase A (10 mg/mL) in 1.5 mL 2×SSC] for 1 h 30min. Next, chromosomes were treated with 0.005% (v/v) pepsin solution [99 µL H_2_O, 10 µL 1M HCl, and 2.5 µL pepsin (20 mg/mL)]. Slides were denatured in 70% formamide in 2× SSC at 72 °C for 3 min. The probe mix per each slide contained 200 ng of each painting probe and 20 µg of blocking DNA which was obtained by DOP-PCR from *H. carvalhoi* genome and used as a blocker to highly and moderated repeated sequences (Yang et al., 2009). The final probe mixture was dissolved in the hybridization buffer containing 50% formamide, 2× SSC and 10% dextran sulfate, then denatured for 10 min at 86°C, cooled at 4°C for 2 min, and pre-annealed for 40 min at 37°C before being applied onto denatured chromosome slides. Hybridization took place in a dark moist chamber at 37°C for 72 h, followed by a series of post-hybridization washes in 1× SSC, 4× SSC/Tween, and 1× PBS solutions (for details, see Sassi et al., 2023). Chromosomes were finally counterstained with 4’, 6-diamidino-2-phenylindole (DAPI) 0.2 μg/mL in Vectashield mounting medium (Vector, California, United States).

### 2.4 Microscopic analyses and image processing

At least 30 metaphase spreads per individual were evaluated to confirm the WCP results. Metaphases were captured in an Axioplan II microscope (Carl Zeiss Jena GmbH, Germany) fluorescence microscope using the ISIS software (MetaSystems, Silver spring, MD, USA).

## 3 Results

**FIGURE 2:**
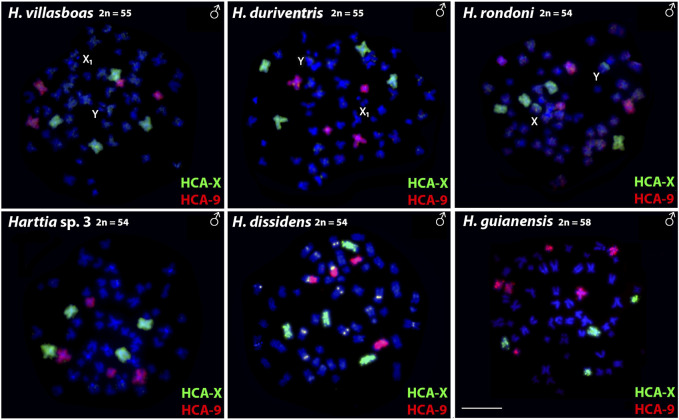
Whole chromosome painting with HCA-X (green) and HCA-9 (red) probes on the male chromosomes of six *Harttia* species. Sex chromosomes are indicated if present and detectable in the given species (first line). Scale Bar = 10 µm.

**FIGURE 3:**
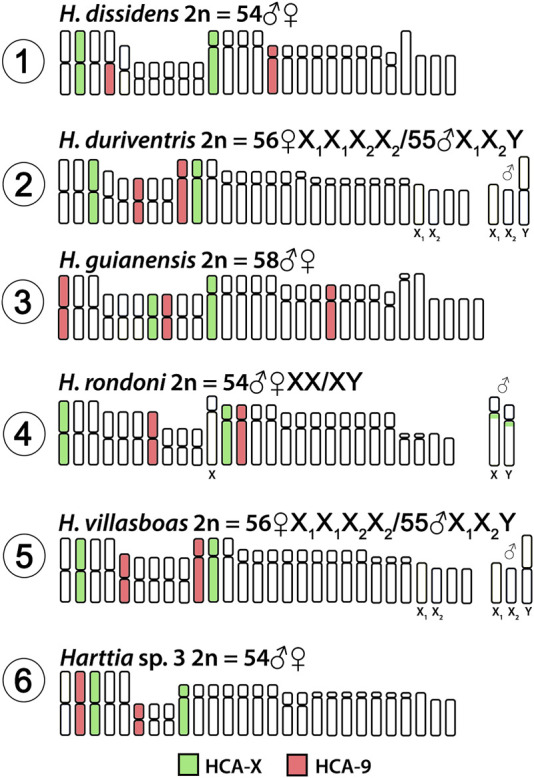
Idiogram summarizing obtained WCP/Zoo-FISH patterns by HCA-X and HCA-9 probes. HCA = *Harttia carvalhoi*.

Both HCA-X and HCA-9 probes hybridized to the chromosomes of all analyzed species (Figure 2). Each probe completely painted from end to end two distinct chromosome pairs (one metacentric and one submetacentric in both cases) in *H. villasboas*, *H. duriventris*, *H. rondoni*, and *Harttia* sp. 3. In addition to those pairs, *H. guianensis* also demonstrated a small metacentric pair probed with HCA-9 (Figure 2). In *H. rondoni*, additional HCA-X signals were found in pericentromeric regions of XY sex chromosomes, probably due to shared ribosomal DNA (rDNA) content which has been amplified on sex chromosomes in this species. Finally, in *H. dissidens*, the HCA-9 probe stained entirely one submetacentric pair but further only long arms of one metacentric pair, while the HCA-X probe revealed the same patterns as for the majority of studied species, with a metacentric and a submetacentric pair being stained. In this species, repetitive DNA sequences contained in both painting probes form distinct clusters, revealed as additional hybridization signals on several chromosomes. An ideogram compiling the present WCP results is provided in Figure 3.

## 4 Discussion

In the present study, we mapped two WCP probes prepared from *H. carvalhoi* chromosomes, where the chromosomes X and 9 were involved in rearrangements leading to emergence of the XY_1_Y_2_ sex chromosome system which is present in three *Harttia* species (Deon et al., 2022a). Both probes painted mostly entire chromosomes, thus pointing to preserved synteny blocks. The probes hybridized only to chromosomes without detectable sex-specific differences in size and shape (and therefore do not represent differentiated sex chromosomes) in species from the Amazonian and Guyanese clades (Figures 2, 3). These findings corroborate the view of independent events behind X_1_X_2_Y and XY_1_Y_2_ origin in *Harttia* (Deon et al., 2022a; b). We also show that chromosome 9 of *H. carvalhoi*, which has been fused to X chromosome in *H. intermontana*, is not involved in any other known sex chromosome system in *Harttia*, however, its propensity to fuse with other chromosomes has been recorded in *H. dissidens*.

The ancestral karyotype of *Harttia* is thought to be 2n = 58, with poorly differentiated or absent sex chromosomes (Blanco et al., 2017; Sassi et al., 2021). Until now, four species – *H. gracilis*, *H. guianensis*, *H. kronei*, *H. longipinna*–from the three phylogenetic clades are known to share these karyotype characteristics. In addition, *H. punctata* exhibits 58 chromosomes in females, but 57 in males due to the presence of X_1_X_2_Y sex chromosome system. Except for *H. guianensis*, which possesses three chromosome pairs labeled with the HCA-9 probe, all remaining five examined species, including *H. punctata* (Deon et al., 2022b), share the pattern of two distinct chromosome pairs being painted either with HCA-X or HCA-9 probe. Based on this evidence, it is possible to infer that the chromosome number reduction from the probable ancestral 2n = 58 to 2n = 56 in *H. loricariformis*, *H. villasboas*, and *H. duriventris*, and to 2n = 54 in *H. rondoni* and *Harttia* sp. 3 was achieved by chromosome fusions which did not involve the herein mapped chromosomes. On the other hand, in *H. dissidens* (2n = 54♀♂, Figure 2) the HCA-9 probe mapped to a submetacentric pair and the short arms of a metacentric pair, thus indicating that a fusion event involving the chromosome 9 of *H. carvalhoi* was responsible for its 2n reduction in relation to the ancestral karyotype. In support of this hypothesis, *H. guianensis* (2n = 58) also possesses more acrocentric chromosomes – five pairs – than the other species with lower 2n (Blanco et al., 2017; Deon et al., 2020; Sassi et al., 2020; Sassi et al., 2021). While the trajectory via centric fusions of acrocentric chromosomes seems highly probable, based on the available data we cannot entirely rule out also the possible involvement of biarmed (metacentric and submetacentric) chromosomes in the (specifically end-to-end/tandem) fusion events (cf. Schubert and Lysak, 2011) in the evolutionary history of *Harttia*.

It is notable that the patterns of hybridization in the Zoo-FISH experiments follows a biogeographical pattern, with *H. guianensis* (distributed in Guyanese drainages) showing three chromosomal pairs labeled with HCA-9, while the species from Amazonian and Tocantins-Araguaia River basins share the pattern of two chromosomal pairs being stained with the same particular WCP probe. Indeed, species from the Amazonian and Tocantins-Araguaia River basins exhibit more similar cytogenetic features when compared to *H. guianensis*, the only species from the Guyanese drainages karyotyped to date (discussed also in Sassi et al., 2021). Based on the cytogenetic and phylogenetic data from nuclear and mitochondrial DNA (Covain et al., 2016), it is possible to suggest that species from Guyanese drainages carry the ancestral-like karyotype arrangement (Sassi et al., 2021). As *H. guianensis* with the most diversified karyotype is the only species from the left bank of the Amazon River, it is tempting to speculate about the possible role of Amazon River as a barrier for gene flow. However, it is difficult to address this issue in the light of the still growing list of *Harttia* species which have been described within the last few years (Oyakawa et al., 2018; Caldas et al., 2022), and the existence of at least three karyotype forms proposed to represent new *Harttia* species (see Deon et al., 2020; Sassi et al., 2021). Moreover, at least seven *Harttia* species are known to occur in Guyanese drainages (Py-Daniel et al., 2001), but only *H. guianensis* has been investigated cytogenetically thus far. It is also noteworthy that the phylogenetic patterns of *Harttia* spp. are not well resolved, with the previously recognized monophyletic status of the genus (Covain et al., 2016) being questioned in a recent study (Cherobim, 2022).

Both the X and Y chromosomes of *H. rondoni* exhibit a positive HCA-X signal in the pericentromeric region of their long arms. As our previous study showed that pericentromeric regions of these chromosomes are enriched with tandem repeats of the major rDNA gene cluster (Sassi et al., 2020), the observed signal pattern might be attributed rather to shared repeat content between the HCA-X probe and the X and Y chromosomes and therefore does not represent homology of synteny blocks. However, the chromosome X of *H. carvalhoi* does not carry major rDNA site, neither do the chromosomes composing both forms of the XY_1_Y_2_ sex system of *Harttia* sp. 1 and *H. intermontana* (Blanco et al., 2017; Deon et al., 2020; 2022a). Bearing also in mind that rDNA sites outside the homeologous synteny blocks were not revealed in other herein studied species by the painting probes, it is highly probable that other repeat class interspersed with rDNA on the XY sex chromosomes of *H. rondoni* is responsible for the observed signal pattern. Analogous situation has been observed after application of HCA-X or HCA-9 probe in other *Harttia* spp. (Deon et al., 2022b). Similarly, an unknown repeat sequence contained in both painting probes demonstrated homology with certain regions on multiple chromosomes of *H. dissidens*.

Sex chromosomes in Loricariidae are promising targets for evolutionary research, since this family is the most diverse among Neotropical fishes (Fricke et al., 2023) and exhibits a wide range of sex chromosomes at distinct stages of differentiation (reviewed in Sember et al., 2021). Taking *Harttia* as an example, although inhabiting large Brazilian rivers, such as São Francisco, Paraná and Araguaia (Caldas et al., 2022), most species are found in riffle areas where they form isolated subpopulations with restricted/absent gene flow. This condition is being further pronounced by low vagility of these fishes, favoring a faster fixation of chromosomal rearrangements by genetic drift (Lande, 1977; King, 1993; Araya-Jaime et al., 2017), and thus also faster species divergence. Structuring into small, geographically isolated populations may also facilitate sex chromosome differentiation and transition between sex chromosome systems (e.g., Primo et al., 2017; Cioffi et al., 2018; Krysanov & Demidova, 2018; Glugoski et al., 2020; Štundlová et al., 2022; Marajó et al., 2023) To answer the question whether indeed genetic drift is a major force in sex chromosomes fixation in *Harttia* and other fish lineages with similar eco-geographical and demographic features and whether certain selective forces and ecological factors (cf. Pennell et al., 2015; Veller et al., 2017; Saunders et al., 2018; Meisel, 2022) might have played a role in this process, we have to acquire much deeper knowledge especially concerning genetic content and degree of sex chromosome differentiation, presence/absence of sex chromosomes in wide range of conspecific populations, and their phylogenetic distribution based on well-resolved phylogeny (cf. Sember et al., 2021). Particularly in *Harttia*, most of the phylogenetic reconstructions do not include all valid species, neither those recognized by cytogenetic studies (Covain et al., 2016; Roxo et al., 2019; Londono-Burbano and Reis, 2021; Sassi et al., 2021; Cherobim, 2022). In this context, given the collection efforts performed within the frame of the recent cytogenetic studies, distinct evolutionary units were identified in *Harttia* (Deon et al., 2020; Sassi et al., 2020) and their recognition as valid species remains to be investigated.

In the present study, we provide another piece of evidence strengthening the proposed view about 2n reduction in *Harttia* by fusions and about independent evolution of *Harttia* multiple sex chromosomes, with repeated origins involving different synteny blocks among species. A thorough phylogenetic reconstruction including all currently recognized representatives of *Harttia* is urgently needed for achieving a complete picture of sex chromosome evolution and karyotype reshaping in this noteworthy group of the Neotropical ichthyofauna.

## Data Availability

The original contributions presented in the study are included in the article/supplementary material, further inquiries can be directed to the corresponding author.
